# Enhanced Recovery After Surgery (ERAS) Reduces Hospital Costs and Improve Clinical Outcomes in Liver Surgery: a Systematic Review and Meta-Analysis

**DOI:** 10.1007/s11605-019-04499-0

**Published:** 2020-01-03

**Authors:** L. Noba, S. Rodgers, C. Chandler, A. Balfour, D. Hariharan, V. S. Yip

**Affiliations:** 1 School of Health in Social Science, University of Edinburgh, Old College, South Bridge, Edinburgh, EH8 9YL UK; 2 Surgical Services, NHS Lothian, Edinburgh, EH1 3EG UK; 3 Hepato-Pancreato-Biliary (HPB) Unit, Royal London Hospital (Barts Health NHS Trust), London, E1 1FR UK

**Keywords:** Enhanced recovery after surgery, Liver surgery, Systematic review and meta-analysis

## Abstract

**Background:**

Enhanced recovery after surgery (ERAS) protocols are evidence-based, multimodal and patient-centred approach to optimize patient care and experience during their perioperative pathway. It has been shown to be effective in reducing length of hospital stay and improving clinical outcomes. However, evidence on its effective in liver surgery remains weak. The aim of this review is to investigate clinical benefits, cost-effectiveness and compliance to ERAS protocols in liver surgery.

**Methods:**

A systematic literature search was conducted using CINAHL Plus, EMBASE, MEDLINE, PubMed and Cochrane for randomized control trials (RCTs) and cohort studies published between 2008 and 2019, comparing effect of ERAS protocols and standard care on hospital cost, LOS, complications, readmission, mortality and compliance.

**Results:**

The search resulted in 6 RCTs and 21 cohort studies of 3739 patients (1777 in ERAS and 1962 in standard care group). LOS was reduced by 2.22 days in ERAS group (MD = −2.22; CI, −2.77 to −1.68; *p* < 0.00001) compared to the standard care group. Fewer patients in ERAS group experienced complications (RR, 0.71; 95% CI, 0.65–0.77; *p* = < 0.00001). Hospital cost was significantly lower in the ERAS group (SMD = −0.98; CI, −1.37 to – 0.58; *p* < 0.0001).

**Conclusion:**

Our review concluded that the introduction of ERAS protocols is safe and feasible in hepatectomies, without increasing mortality and readmission rates, whilst reducing LOS and risk of complications, and with a significant hospital cost savings. Laparoscopic approach may be necessary to reduce complication rates in liver surgery. However, further studies are needed to investigate overall compliance to ERAS protocols and its impact on clinical outcomes.

## Introduction

The concept of developing a ‘multimodal approach’ to accelerate recovery and rehabilitation after surgery was first developed by Kehlet.^1^ This approach later evolved into what is now known as enhanced recovery after surgery (ERAS). ERAS protocols have been implemented in various surgical specialties especially colorectal surgery from the early 2000s. However, due to patient safety concerns and high complication rates, the first cases of ERAS protocols in liver surgery only appeared in the scientific journals in 2008.^2, 3^

The ERAS programme is an evidence-based, multimodal and patient-centred approach to optimize patient care and experience during perioperative care.^4^ In 2016, the ERAS Society published its first guideline for perioperative care in liver surgery to add to existing ERAS guidelines regarding other surgical specialities.^5^ This particular guideline consists of 23 items including preoperative counselling, preoperative carbohydrate loading, perioperative nutrition, avoidance of bowel preparation, no routine use of surgical drain, thromboembolism prophylaxis, antibiotic prophylaxis, minimally invasive approach, intraoperative fluid restriction, multimodal analgesia, prevention of hypothermia, early oral fluid and normal diet intake, glycaemic control, prevention of delayed gastric emptying, stimulation of bowel movement, early mobilization, prevention of postoperative nausea and vomiting, fluid management and systematic audit. Majority of the recommendations were based on evidence from colorectal surgery due to limited evidence in liver surgery. With entirely different patient cohorts between colorectal and liver surgery, their different morbidities and therefore physiological stress on patients, it is questionable whether ERAS principles used in colorectal surgery can be truly extrapolated to liver surgery.

Traditionally, liver surgery is known to be associated with high complication and mortality rates.^6^ With recent advancement in surgical techniques and improvement in perioperative care management, a mortality rate of less than 5% is now achievable.^7^ Moreover, several reviews concluded that the implementation of ERAS protocols is associated with a significant reduction in length of hospital stay and postoperative complications without increasing mortality and readmission rates.^7–14^ However, evidence on cost-effectiveness and compliance to ERAS protocols in liver surgery is limited. Two previous meta-analyses have demonstrated a significant reduction in hospital expenditure following implementation of ERAS protocols in liver surgery.^9, 10^ The conclusion reached by these reviews was either based on low-quality RCTs and cohort studies without conducting a separate meta-analysis of RCTs and cohort studies. Furthermore, to date, no review has investigated overall protocol compliance which is a key factor to delivering a successful implementation of ERAS protocols. The aim of this review is to investigate clinical benefits, cost-effectiveness and compliance to ERAS protocols in liver surgery.

## Method

### Search Strategy

This review was conducted in accordance with PRISMA (preferred reporting items for systematic reviews and meta-analyses) guidelines for meta-analysis.^15^ A systematic search was conducted in March 2019 on the following database: CINAHL Plus, EMBASE, MEDLINE, PubMed and Cochrane for studies published between 2008 and 2019. The search was limited to English language. Reference lists of relevant RCTs and systematic reviews were searched for eligible studies. The search terms such as ‘enhanced recovery’, ‘fast track’, ‘ERAS’, ‘perioperative care’, ‘enhanced rehabilitation’, ‘liver resection’, ‘liver surgery’, ‘hepatic resection’, ‘hepatobiliary’, ‘HPB surgery’, ‘hepato-pancreato-biliary’ and ‘hepatectomy’ were applied using Boolean operator (OR and AND).

### Inclusion/Exclusion Criteria

RCTs and non-RCTs studies were eligible for inclusion if they all meet the following criteria, (1) studies of adult patients undergoing liver surgery, (2) compared ERAS to standard care and (3) report on at least one of the following outcomes: length of hospital stay (LOS), complication rate, hospital cost, readmission rates and mortality rates. Studies were excluded if they were non-elective or transplant patients, non-English and not comparing ERAS to standard care.

### Data Extraction

All potential studies and relevant data were retrieved and extracted by one author (LN). Data were extracted using a data extraction sheet agreed by all reviewers, and data extraction was subsequently validated by other authors. Data extracted included authors’ first and last names, study design, patient’ characteristics (ASA grade, age and sex), type of surgery, outcomes measured, sample size, follow-up period and ERAS programme items.

### Literature Search

**Fig. 1:**
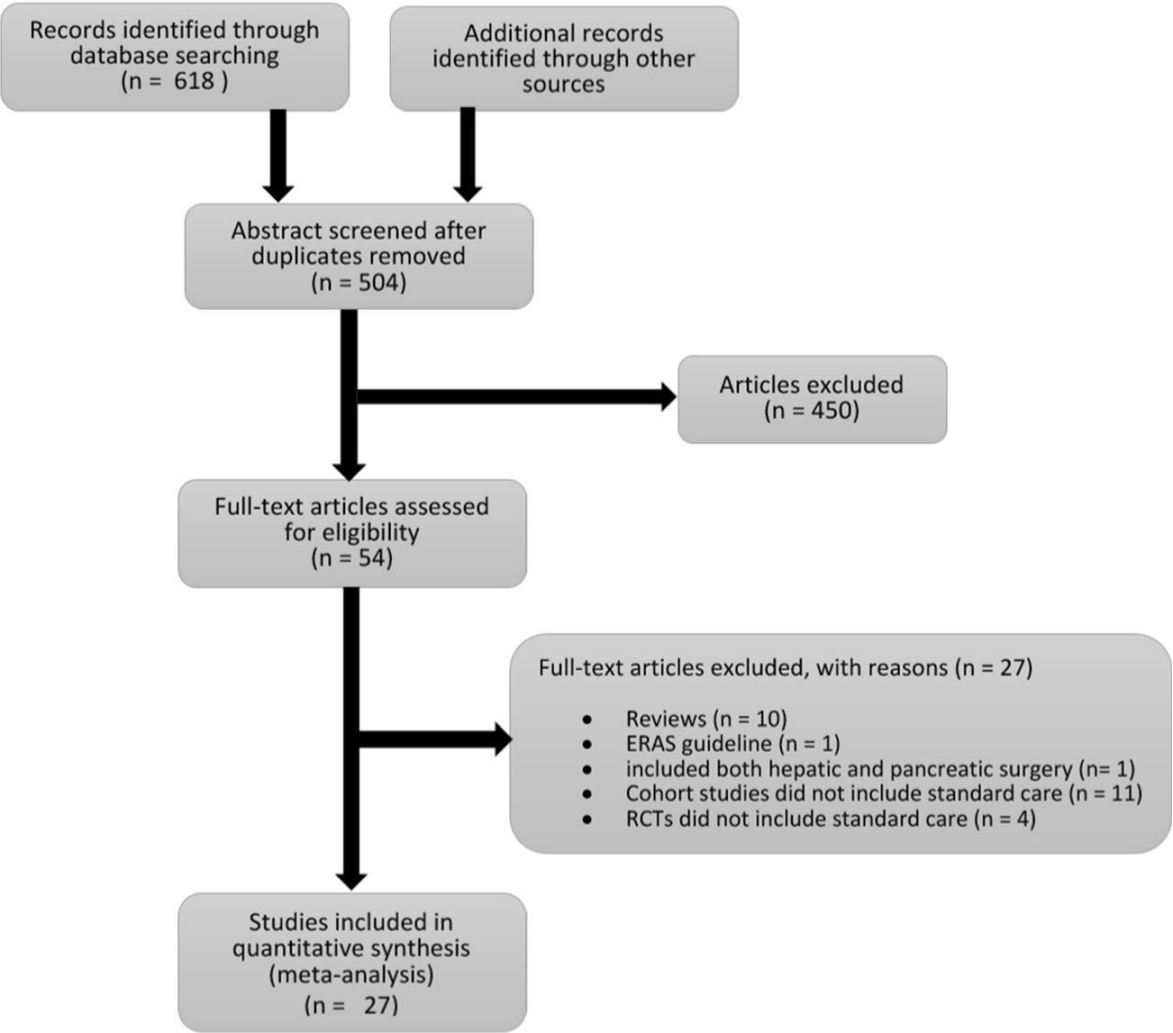
Flow chart of study selection process

An initial search yielded 631 studies. Five hundred four abstracts were screened after duplicates were removed. Four hundred fifty ineligible articles identified through screening were removed. Fifty-four full-text articles were assessed for eligibility, of which *27* were excluded for the following reasons: 1 study included both hepatic and pancreatic surgery, *11* cohort studies did not include standard care, 4 RCTs did not include standard care, 1 paper related only to ERAS guidelines and 10 were reviews. The final *27* studies were included in the meta-analysis (Fig. 1).

### Outcomes of Interest

The primary outcome for this systematic review was hospital cost. Secondary outcomes include LOS, complication rate, readmission rates, mortality rates and compliance.

### Quality Assessment

**Fig. 2:**
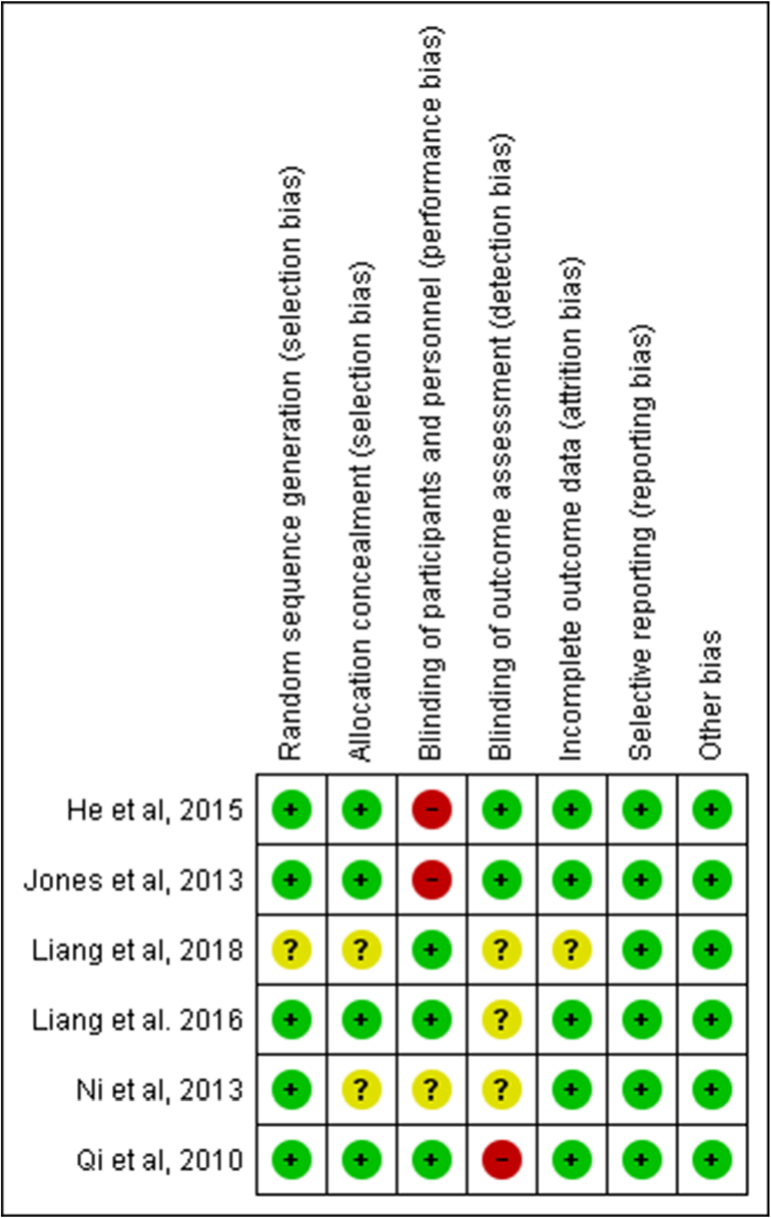
Summary of risk of bias of RCTs

The quality of the RCTs was assessed in accordance with the Cochrane Collaboration’s risk of bias tool^16^ (Fig. 2). The Modified Downs and Black checklist was used for the assessment of the methodological quality of both randomized and non-randomized studies for both RCTs and non-RCTs.^17^ The Modified Downs and Black checklist has a maximum of 28 points (11 points for reporting, 3 points for external validity, 7 points for internal validity – bias, 6 points for internal validity – confounding (selection bias) and 1 point for power calculation (Table 1).

**Table 1:** Characteristics of included studies

Studies	Year	Country	Study design	Surgery approach	ASA grade		Age		Sex			Sample size		Quality of study score
				ERAS	CS	ERAS	CS	ERAS -	CS -	ERAS	CS			
					I/II/III/IV	I/II/III/IV			M/F		M/F			
Clark et al.^18^	2016	USA	Case-control	Open	1/29/23/0	3/42/28/0	59 (47–68)	65 (51–73)	22/31		36/37	53	73	18
Dasari et al.^19^	2015	UK	Prospective study	Lap/open	x	x	65.2 (12.3)	63.4 (10.8)	58/33		58/35	91	93	20
Day et al.^20^	2015	USA	Prospective study	Lap/open	2(I/II/IV)/ 73(II/IV)	3(I/II/IV)/40(III/IV)	59 (48–97)	60 (47–67)	47/28		18/25	75	43	19
Ding et al. (lap)^21^	2018	China	Case-control	Lap	x	x	56.04 ± 11.50	56.31 ± 11.57	31/18		88/45	49	133	16
Ding et al. (open)^21^	2018	China	Case-control	Open	x	x	57.10 ± 10.42	58.95 ± 10.89	12/8		76/23	20	99	16
He et al.^22^	2015	China	RCT	Lap	10/26/2/0	12/24/2/0	56.3 ± 16.3	60.4 ± 20.7	22/26		18/202	48	38	21
Jing et al.^23^	2018	China	Case-control	Lap/open	1/53/25/0	0/88/33/0	55.47 ± 11.26	56.92 ± 10.89	52/27		85/36	79	121	20
Joliat et al.^24^	2016	Switzerland	Case-control	Lap/open	56 (I-II)/18/0	72(I-II)/28/0	60.5 (50–68.25)	64 (57.25–69.75)	43/31		60/40	74	100	19
Jones et al.^25^	2013	UK	RCT	Open	0/43/3/0	2/38/5/0	64 (27–83)	67 (27–84)	31/15		23/22	46	45	23
Kaibori et al.^26^	2017	Japan	Prospective study	Open	X	X	71 (60–81)	69 (61–77)	37/10		22/2	47	24	19
Labgaa et al. ^27^	2016	Switzerland	Case-control	Lap/open	56(I-II)/18(III-IV)	36 (I-II)/ 14(III-IV)	60 (49.75–68)	64 (59–69)	43/31		35/15	74	50	20
Liang et al.^28^	2018	China	RCT	Lap	12/35/11	8/48/5/0	58 (16–80)	59 (37–85)	33/25		39/22	58	61	27
Liang et al.^29^	2016	China	RCT	Lap	35/45/0/0	49/58/0/0	53.4 (±13.5)	55.5 (±12.8)	37/43		50/57	80	107	22
Lin et al.^30^	2011	China	Prospective study	Open	43 (I-II)/11/2	50 (I-II)/10/1	57(23–73)	55(22–81)	31/25		34/27	56	61	18
Ma et al.^31^	2018	China	Case-control	Open	37/11/26/0	37/11/26/0	46.1 ± 10.6	45.2 ± 11.7	65/9		64/10	74	74	20
Ni et al.^32^	2013	China	RCT	Open	76/4/0/0	78/2/0/0	48.4 ± 15.6	50.1 ± 21.8	66/14		59/21	80	80	22
Ovaere et al.^33^	2017	Belgium	Case-control	Lap/open	8/25/17/0	19/24/7/0	64.5 (56.0–73.0)	63.0 (51.0–74.0)	26/24		19/31	50	50	18
Page et al.^34^	2016	USA	Prospective study	Open	x	x	60 (48–68)	54 (47–68)	41/34		21/21	75	42	18
Qi et al.^35^	2018	China	RCT	Lap/open	0/10/60/x	0/12/68/0	53.7 ± 9.8	55.4 ± 9.2	47/33		40/40	80	80	26
Sánchez-Pérez et al.^36^	2012	Spain	Case-control	Lap	0/13/13/0	0/9/8/0	58.3 (29–77)	52.5 (29–84)	15/11		10/7	26	17	15
Savikko et al.^37^	2015	Finland	Case-control	Lap/open	3/42/78/11	2/28/63/7	63 (26–86)	65 (18–84)	78/56		55/45	134	100	17
Stoot et al.^38^	2009	Netherlands	Case-control	Lap	03/9/1/0	06/06/1/0	55 (34–82)	45 (26–70)	3/10		2/11	13	13	17
Sutherasan et al.^39^	2017	Thailand	Case-control	Lap/open	46/106/13/0	51/118/13/0	55.71 (±11.32)	58.33 (±11.73)	106/59		123/59	165	182	18
Teixeira et al.^40^	2019	Brazil	Case-control	Lap/open	x	x	58 (24–78)	60 (22–82)	16/19		22/28	35	50	13
Thornblade et al.^41^	2018	USA	Case-control	Open	1/22/42/4	1/11/41/5	56.0 (13.6)	52.9 (12.1)	37/32		34/24	69	58	17
Van Dam et al.^2^	2008	UK	Case-control	Open	11/42/8/0	14/64/22/0	62 (24–82)	60 (20–81)	35/26		51 /49	61	100	20
Zhu et al.^42^	2014	China	Case-control	Open	X	x	53(32–71)	52(30–73)	40/25		46/22	65	68	17

*Quality of study score according to Downs and Black checklist (1–28).

## Statistical Analysis

This meta-analysis was performed using Review Manager (RevMan) version 5.3.^43^ Relative risk was used for all dichotomous variables, weight mean difference or weight standardized mean difference for continuous variable with 95% confidence interval.^44^ Statistical significance level was set at *p* < 0.05. Statistical heterogeneity was assessed using a chi-squared test (χ2), I^2^ statistic, with *p* < 0.1 considered to be statistically significant. A fixed effect model was used for pooling. Where there is significant evidence of heterogeneity (> 50%), random effect model was used instead. Using the method developed by devised Hozo, Djulbegovic and Hozo (2005), study data presented as medians and ranges or medians and interquartile ranges were converted to mean and standard deviation (SD). Funnel plots were used to assess presence of publication bias.

## Results

### Characteristics of Included Studies

A total of 3739 patients were included in the review (ranging between 26 and 347 per study), 1777 were managed according to an ERAS protocol and 1962 according to standard perioperative care. The ERAS elements utilized in the studies range between 8 and 23. A detailed list of ERAS elements utilized by each study is shown in Table 2. Six studies were RCTs,^22, 25, 28, 29, 32, 35^ 16 were case-control studies^2, 18, 21, 23, 24, 27, 31, 33, 36–42^ and five prospective studies.^19, 20, 26, 30, 34^ Eleven studies included both open and laparoscopic surgery in the study,^19–21, 23, 24, 27, 33, 35, 37, 39, 40^ ten studies included only open liver surgery patients,^2, 18, 25, 26, 30–32, 34, 41, 42^ five studies included only laparoscopic surgery patients,^22, 28, 29, 36, 38^ whilst one study performed a separate analysis for laparoscopic surgery and open surgery.^21^ A detailed characteristic of included studies is shown in Table 1.

**Table 2:** Summary of ERAS elements

Period	ERAS items	Teixeira^40^	Jing^23^	Ding^21^	Ma^31^	Thornblade^41^	Qi^35^	Liang^28^	Kaibori et al.^26^	Ovaere^33^	Sutherasan^39^	Joliat^24^	Page^34^	Clark^18^
Pre-op	Preoperative education	✓	✓	✓	✓	✓	✓	✓	X	✓	X	✓	✓	✓
	Preoperative nutrition	✓	x	✓	X	✓	✓	✓	X	X	X	X	X	X
	No preanesthetic medication	✓	X	X	✓	X	x	X	✓	X	X	X	X	X
	No bowel preparation	✓	✓	X	✓	X	✓	✓	✓	X	✓	✓	X	✓
	Minimal pre-op fasting	✓	✓	✓	✓	✓	✓	✓	✓	✓	X	✓	✓	X
	Carbohydrate drinks 2 h before surgery	✓	X	X	✓	✓	✓	✓	Up to 3 h prior to surgery	✓	X	✓	✓	X
	Thromboembolism prophylaxis	✓	X	X	X	✓	✓	✓	X	X	X	✓	X	✓
Intra-op	Thoracic epidural anaesthesia	✓	✓	X	✓	X	✓	X	✓	✓	✓	✓	✓	X
	Antibiotic prophylaxis	✓	X	X	✓	✓	X	X	X	✓	X	✓	X	X
	Short-acting anaesthetic agent	✓	X	X	X	X	X	✓	✓	✓	X	X	X	X
	Avoid excessive i.v. fluids	✓	✓	X	X	✓	✓	✓	✓	✓	X	✓	✓	✓
	No routine use of NG tubes	✓	X	✓	✓	X	✓	✓	✓	✓	✓	✓	✓	✓
	No routine use of abdominal drain	✓	✓	X	✓	X	✓	✓	✓	✓	✓	✓	✓	✓
	Prevention of hypothermia	✓	✓	X	✓	✓	✓	✓	✓	X	✓	✓	x	X
Post-op	Removal of urinary catheter	X	✓	POD 1	X	X	12 h post-op	POD 1	POD 3	POD 1	POD 2	POD 3	x	POD 1
	Removal of abdominal drains	X	POD 2–3	X	POD 2	X	✓	<POD3	x	POD 2	x	X	X	x
	Post-op laxatives	X	X	X	X	X	X	X	X	X	X	✓	X	X
	Restart water intake	POD 1	POD 1	6 h post-op	POD 1	POD 0	6 h post-op	6 h post-op	POD1	X	X	4 h post-op	x	POD 0
	Restart normal diet	POD 1	✓	POD 1	POD 3	POD 1	POD 1	POD 1	POD 1	POD 1	POD 1	POD 1	x	POD 0
	Early mobilization	✓	✓	POD 1	POD 1	POD 1	POD 1	POD 1	POD1	POD 0	X	POD 0	X	POD O
	Oral analgesia	X	X	X	X	POD 2	X	POD 3	POD 1	X	X	X	X	POD 0
	Ileus prevention	X	X	X	✓	POD 1	X	X	X	X	X	X	X	X
	Glycaemic control	✓	X	X	X	✓	X	X	X	X	X	X	X	X
	Prevention of PONV	✓	X	X	X	X	X	✓	X	X	X	✓	X	✓
	Reduction of IV fluids	✓	X	POD 1	POD 1	POD 1	X	POD 0	POD 1	X	X	POD 1	x	POD 1
	Systemic audit	✓	X	X	X	X	X	✓	X	X	X	✓	X	X
Period	ERAS items	Liang^29^	Labgaa^27^	Savikko^37^	He^22^	Dasari^19^	Day et al.^20^	Zhu et al.^42^	Ni^32^	Jones^25^	Sánchez-Pérez^36^	Lin^30^	Stoot^38^	Van Dam^2^
Pre-op	Preoperative education	✓	✓	X	✓	✓	✓	✓	✓	✓	X	✓	✓	✓
	Preoperative nutrition	✓	X	X	X	X	X	X	X	✓	X	X	X	X
	No preanesthetic medication	X	X	X	X	✓	X	X	✓	✓	X	✓	✓	✓
	No bowel preparation	✓	✓	X	X	X	✓	✓	✓	✓	X	✓	X	X
	Minimal pre-op fasting	✓	✓	X	✓	✓	✓	X	✓	✓	X	✓	✓	✓
	Carbohydrate drinks 2 h before surgery	✓	✓	✓	✓	✓	x	X	✓	✓	X	✓	✓	✓
	Thromboembolism prophylaxis	X	✓	X	X	✓	X	X	X	✓	X	✓	X	x
Intra-op	Thoracic epidural anaesthesia	X	✓	✓	✓	✓	✓	X	✓	✓	X	✓	✓	✓
	Antibiotic prophylaxis	✓	✓	X	✓	✓	X	X	X	✓	X	✓	X	X
	Short-acting anaesthetic agent	X	X	X	✓	X	X	X	X	X	X	X	✓	✓
	Avoid excessive i.v. fluids	✓	✓	X	✓	X	✓	X	✓	✓	✓	X	✓	✓
	No routine use of NG tubes	✓	✓	X	✓	✓	✓	✓	✓	✓	✓	✓	✓	✓
	No routine use of abdominal drain	✓	✓	✓	✓	✓	✓	X	✓	✓	✓	✓	✓	✓
	Prevention of hypothermia	✓	✓	X	✓	✓	X	X	X	✓	X	X	X	✓
Post-op	Removal of urinary catheter	POD 1	POD 1	POD 2–3	POD 1	POD 2	X	X	POD 2	POD 2	X	POD 1	✓	POD 3
	Removal of abdominal drains	POD 2	N/A	POD 1	X	N/A	x	POD 3	POD 2	POD 2	24–48 h post-op	N/A	N/A	N/A
	Post-op laxatives	X	✓	✓	✓	X	X	X	POD 1	X	X	X	X	X
	Restart water intake	6 h post-op	4 h post-op	POD 0	4 h post-op	POD 1	POD 0	POD 1	POD 1	POD 0	6–8 h post-op	6 h post-op	POD 1	POD 0
	Restart normal diet	POD 1	POD 1	POD 0	12 h post-op	POD 1	POD 1	POD 1	POD 3	POD 0	X	6 h post-op	✓	POD 1
	Early mobilization	POD 1	POD 1	POD 0	POD 1	Full mobilization POD 3	POD 1	POD 1	POD 1	POD 1	6–8 h post-op	POD 1	✓	POD 1
	Oral analgesia	POD 2	POD 3	POD 0	X	POD 1	POD 1	POD 4	X	POD 2	POD 2	POD 4	X	POD 1
	Ileus prevention	X	X	X	X	X	X	X	X	X	X	X	X	X
	Glycaemic control	X	X	X	✓	X	X	X	X	✓	X	X	X	x
	Prevention of PONV	X	✓	X	✓	✓	✓	X	x	✓	X	X	X	x
	Reduction of IV fluids	POD 0	POD 1	X	POD 2	POD 1	x	POD 1	x	POD 1	X	POD 1	X	POD 1
	Systemic audit	X	✓	X	X	X	✓	X	X	X	x	X	X	x

### Sensitivity Analysis and Publication Bias

**Fig. 3:**
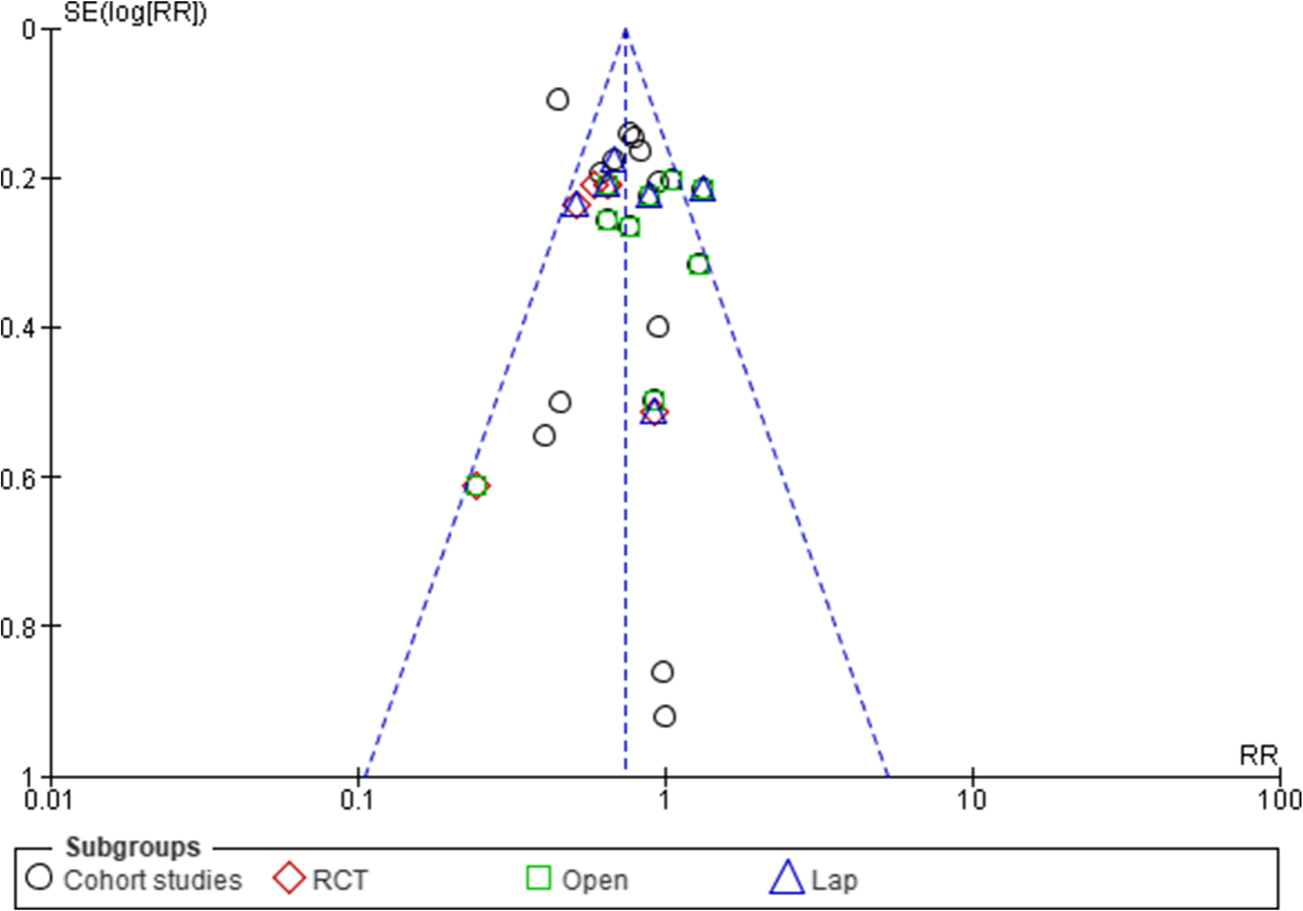
Funnel plots for complication rates

**Fig. 4:**
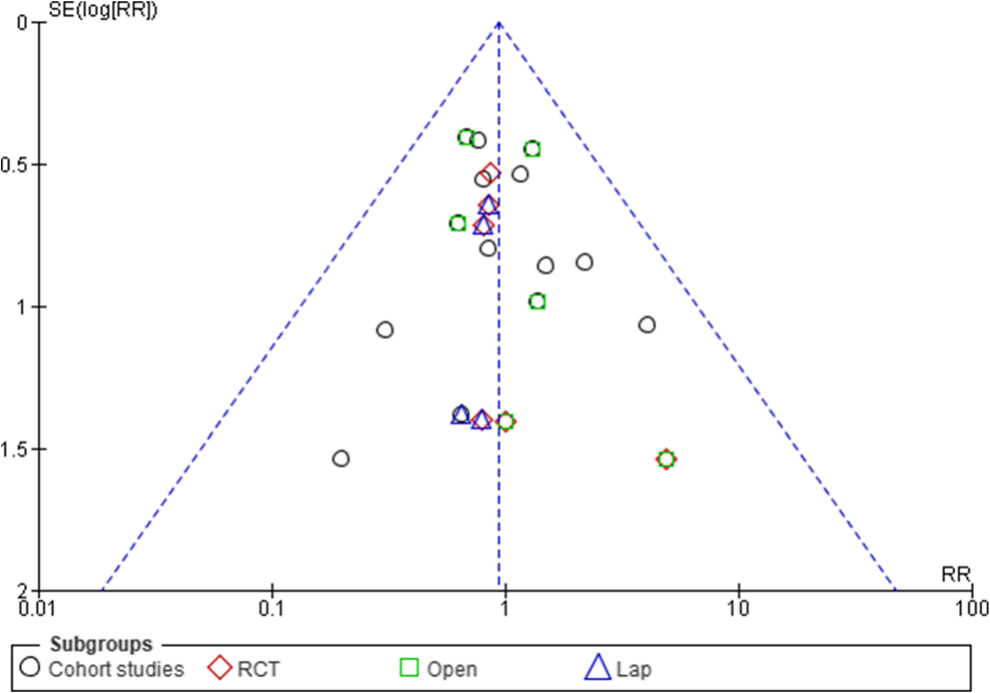
Funnel plots for readmission rates

Funnel plots for overall complication and readmission rates were used to assess publication bias (Figs. 3 and 4). The asymmetry of the funnel plots suggested no evidence of publication bias. Where there was evidence of heterogeneity, a sensitivity analysis was performed to test the reliability of the results.

### Hospital Cost

Twelve studies provided data on hospital cost (1606 patients). Five of the studies reported hospital cost in US dollar,^21, 22, 29, 35, 41^ three in Chinese yuan^28, 30, 42^ and two in euros.^24, 33^ One study reported 30% reduction in hospital cost in ERAS group without providing further data,^36^ and one did not provide data on overall hospital cost.^34^ These two studies were therefore excluded from the meta-analysis.

A 40·7% reduction in laboratory-associated costs ($333), 54·1% in pharmacy-related costs ($332; *p* < 0·001), 21·5% in medical supply costs ($394; *p* = 0·007) and significant reduction in therapy-related costs *(p* < 0·001) were reported in the ERAS group, with no differences between the two groups in operating room or radiology costs. One study reported the hospital cost in mean without standard deviation.^30^ Using a method suggested by Furukawa et al.,^44^ standard deviation was borrowed from a study with similar sample size and mean.^41^

**Fig. 5:**
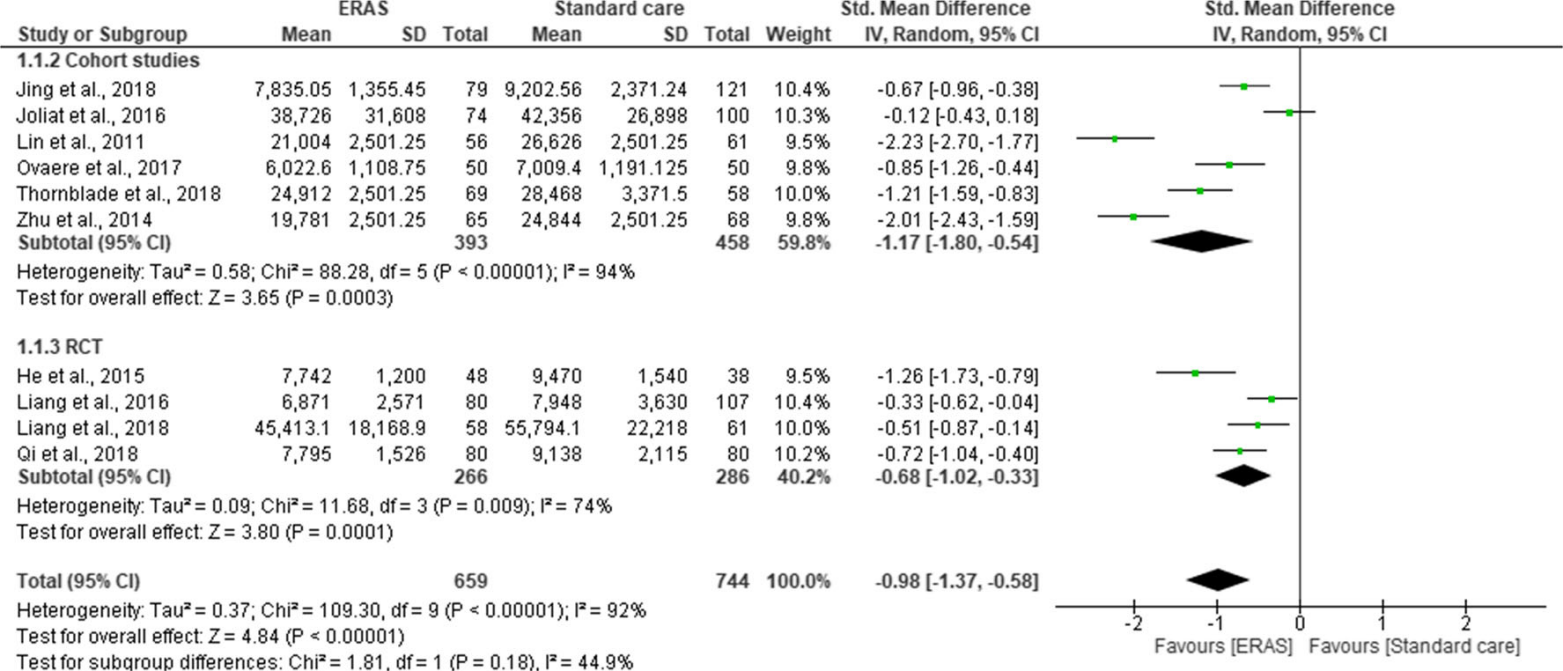
Forest plot of hospital cost, ERAS vs standard care; subgroup (RCT & Cohort studies)

**Fig. 6:**
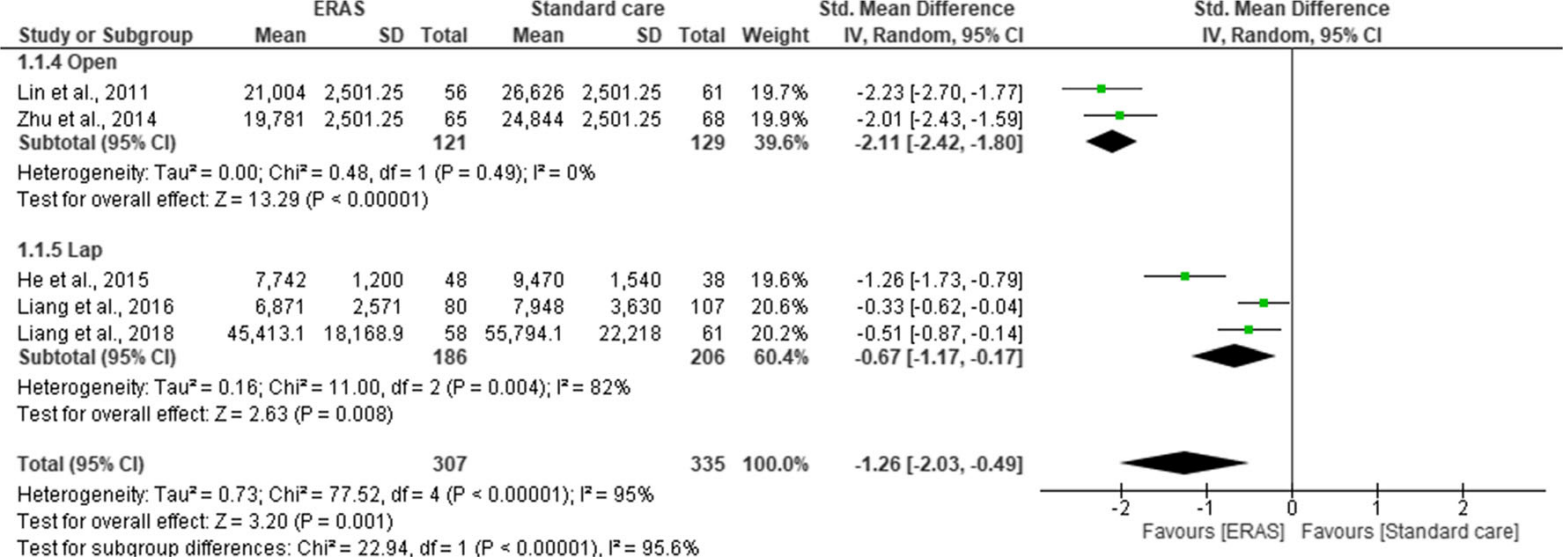
Forest plot of hospital cost, ERAS vs standard care; subgroup analysis (Open & Lap)

Pooling of all results revealed a lower hospital cost in ERAS group compared to standard care (SMD = −0.98; CI, −1.37 to –0.58; *p* < 0.0001). However, there was a significant heterogeneity observed among all studies (χ^2^ = 109.63; df = 9; *p* < 0.0001; I^2^ = 92%). In the subgroup analysis for the type of studies, hospital cost was significantly lower in the ERAS group for both RCTs (SMD = −0.68; CI, −1.02 to –0.33; *p* < 0.0001) and cohort studies (SMD = −1.17.; CI, −1.80 to –0.54; *p* < 0.0003). There was no significant difference in the overall hospital costs between the RCTs and cohort studies (χ^2^ = 1.82; df = 1; *p* < 0.18; I^2^ = 45%) (Fig. 5). Further subgroup analysis for the type of surgical approach confirmed a significant hospital cost savings for both open surgery (SMD = 2.11; CI, 2.42 to −1.80; *p* < 0.00001) and laparoscopic surgery (SMD = 0.67; CI, −1.17 to −0.17; *p* = 0.008) in the ERAS group over standard care. However, there was a significant difference in the overall hospital costs between the two surgical approaches (χ^2^ = 22.94, df = 1 (*p* < 0.00001), I^2^ = 95.6%) (Fig. 6).

### Length of Hospital Stay

**Fig. 7:**
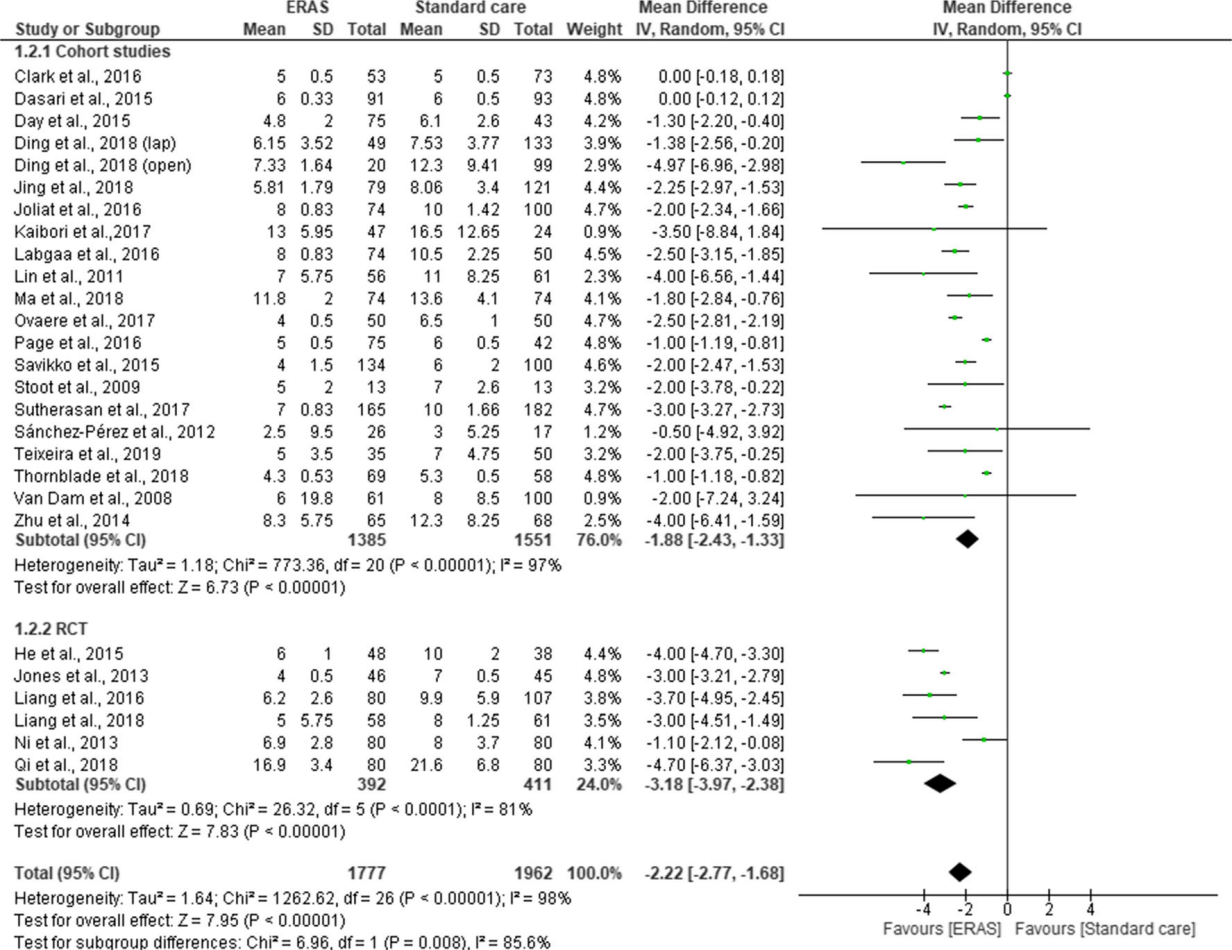
Forest plot of length of hospital stay, ERAS vs standard care; subgroup analysis (RCT & Cohort studies)

**Fig. 8:**
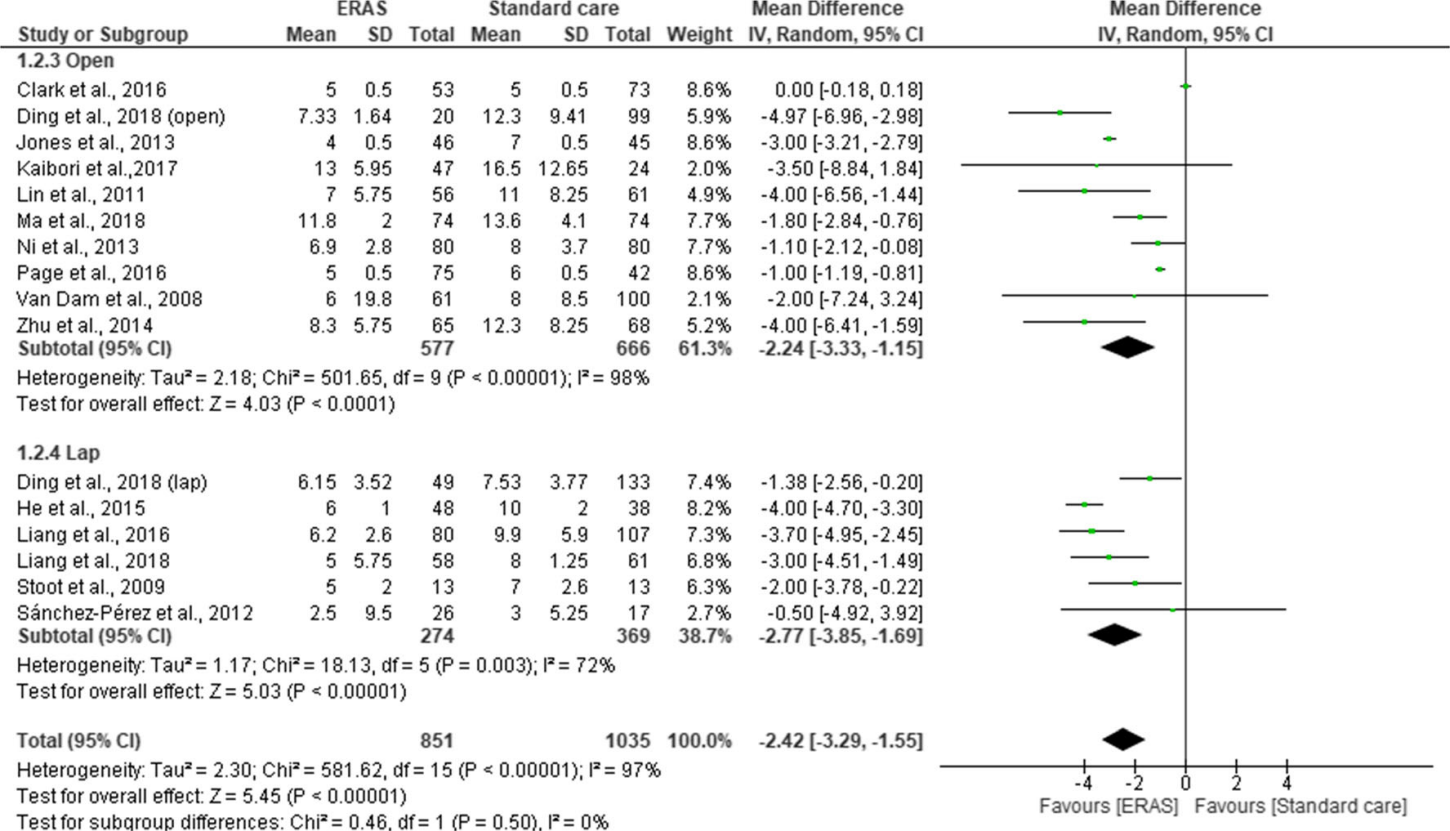
Forest plot of length of hospital stay, ERAS vs standard care; subgroup analysis (Open & Lap)

All the studies reported total length of hospital stay. Pooling the results, LOS was significantly shorter in ERAS group compared to standard care (MD = −2.22; CI, −2.77 to –1.68; *p* < 0.00001), with a significant heterogeneity observed in the studies (χ^2^ = 1262.62; df = 26*; p* < 0.00001; I^2^ = 98%). A subgroup analysis for RCT (MD = −3.18; CI, −3.97 to −2.38; *p* < 0.00001) and cohort studies (MD = −1.88; CI, −2.43 to −1.33; *p* < 0.00001) demonstrated a significant shorter LOS in the ERAS vs standard care group. However, there was a significant difference in overall LOS between RCTs and cohort studies (χ^2^ = 6.69; df = 1; *p* < 0.008; I^2^ = 85.6%) (Fig. 7). Further subgroup analysis for the type of surgical approach found a significant reduction in LOS for both open surgery (MD = −2.24; CI, −3.33 to −1.15; *p* < 0.0001) and laparoscopic surgery (MD = −2.77; CI, −3.85 to −1.69, *p* < 0.00001) in the ERAS group over standard care. There was no significant difference in overall LOS between open surgery and laparoscopic approach (χ^2^ = 0.46, df = 1 (*p* = 0.50), I^2^ = 0%) (Fig. 8).

### Complication Rates

Twenty-six studies (3496 patients) reported overall complication rates, with a total of 1332 patients experiencing complications (507 in ERAS group and 825 in standard care group). One study did not provide data on the number of cases of complication^34^ and was excluded from the meta-analysis.

**Fig. 9:**
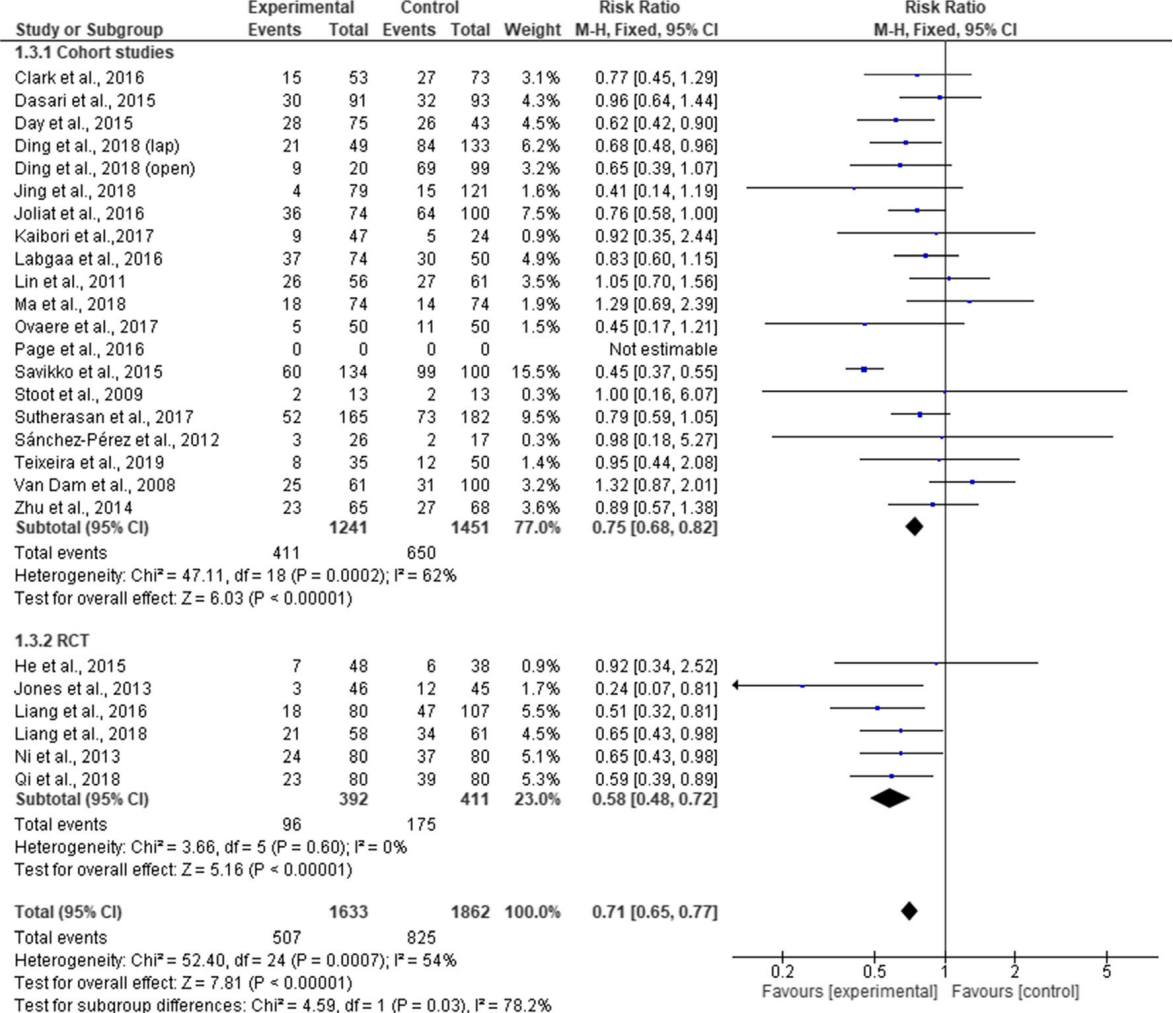
Forest plot of complication rates, ERAS vs standard care; subgroup analysis (RCT & Cohort studies)

**Fig. 10:**
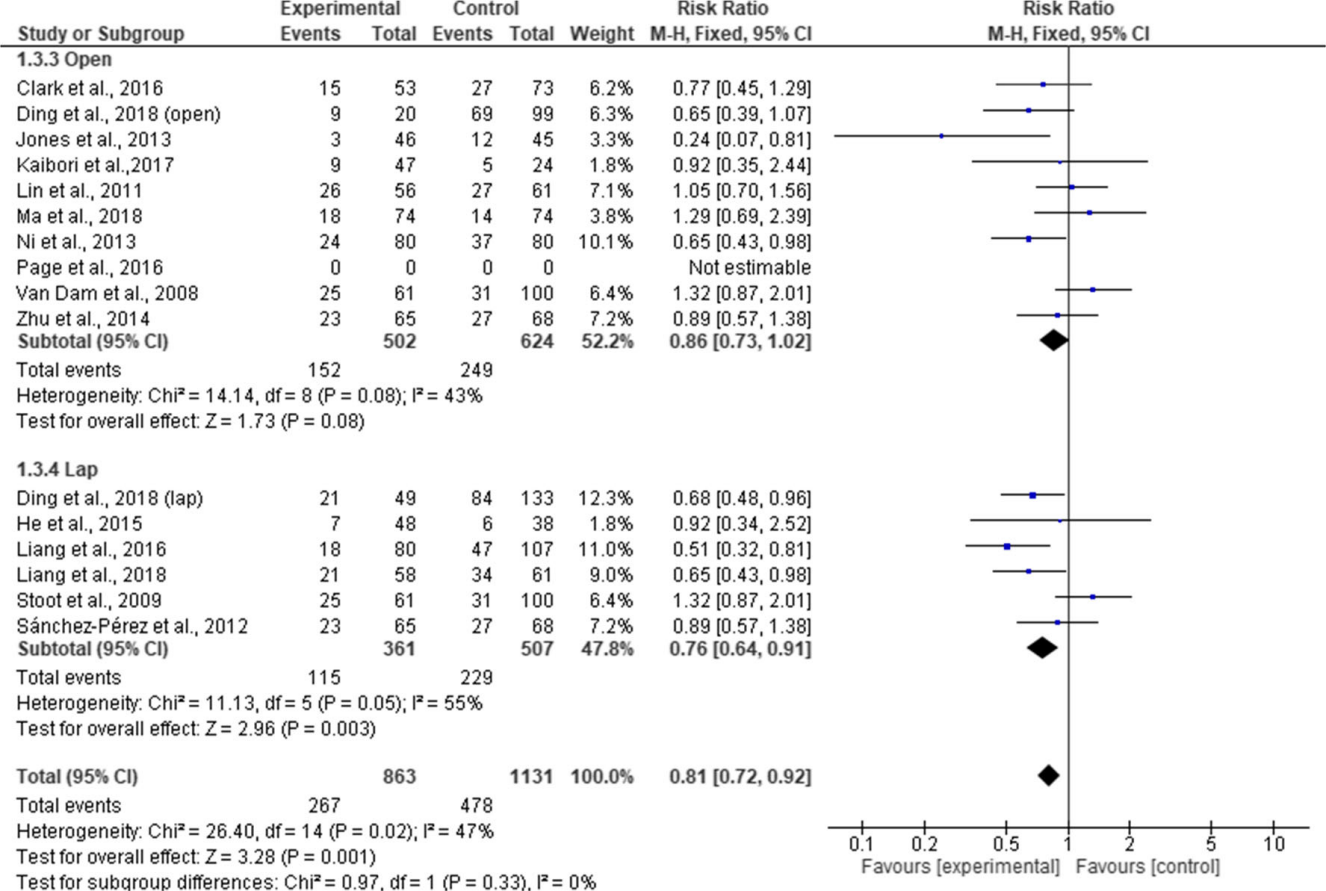
Forest plot of complication rates, ERAS vs standard care; subgroup analysis (Open & Lap)

Pooling the results, there was a significant difference in overall complication rates between the ERAS group and the standard care group (RR, 0.71; 95% CI, 0.65–0.77; *p* = < 0.00001) but with a significant heterogeneity observed in the studies (χ^2^ = 52.50; df = 24; *p* = 0.0007; I^2^ = 54%). In the subgroup analysis, cases of overall complications were significantly less among the ERAS group than standard care in the RCTs (RR, 0.58; 95% CI, 0.48–0. 72; *p* = < 0.00001), cohort studies (RR, 0.75; 95% CI, 0.68–0.82; *p* = < 0.00001). There was a significant difference between RCTs and cohort studies (χ^2^ = 4.59; df = 1; *p* = 0.03; I^2^ = 78.2) **(**Fig. 9). Further subgroup analysis for the type of surgical approach revealed fewer complication rates in the ERAS group compared to standard care for laparoscopic surgery (MD = 0.76; CI, 0.64 to 0.91; *p* = 0.003). However, complication rates in ERAS group and standard care were similar for open surgery (MD = 0.86; CI, 0.73 to 1.02; *p* = 0.08). There was no significant difference in the overall complication rates between the two surgical approaches (χ^2^ = 0.97, df = 1 (*p* = 0.33), I^2^ = 0%). Moreover, Page et al.^34^ found significant fewer incidence of postoperative complications in the ERAS group (1% vs 10%; *p* = 0.036) (Fig. 10).

### Readmission Rates

**Fig. 11:**
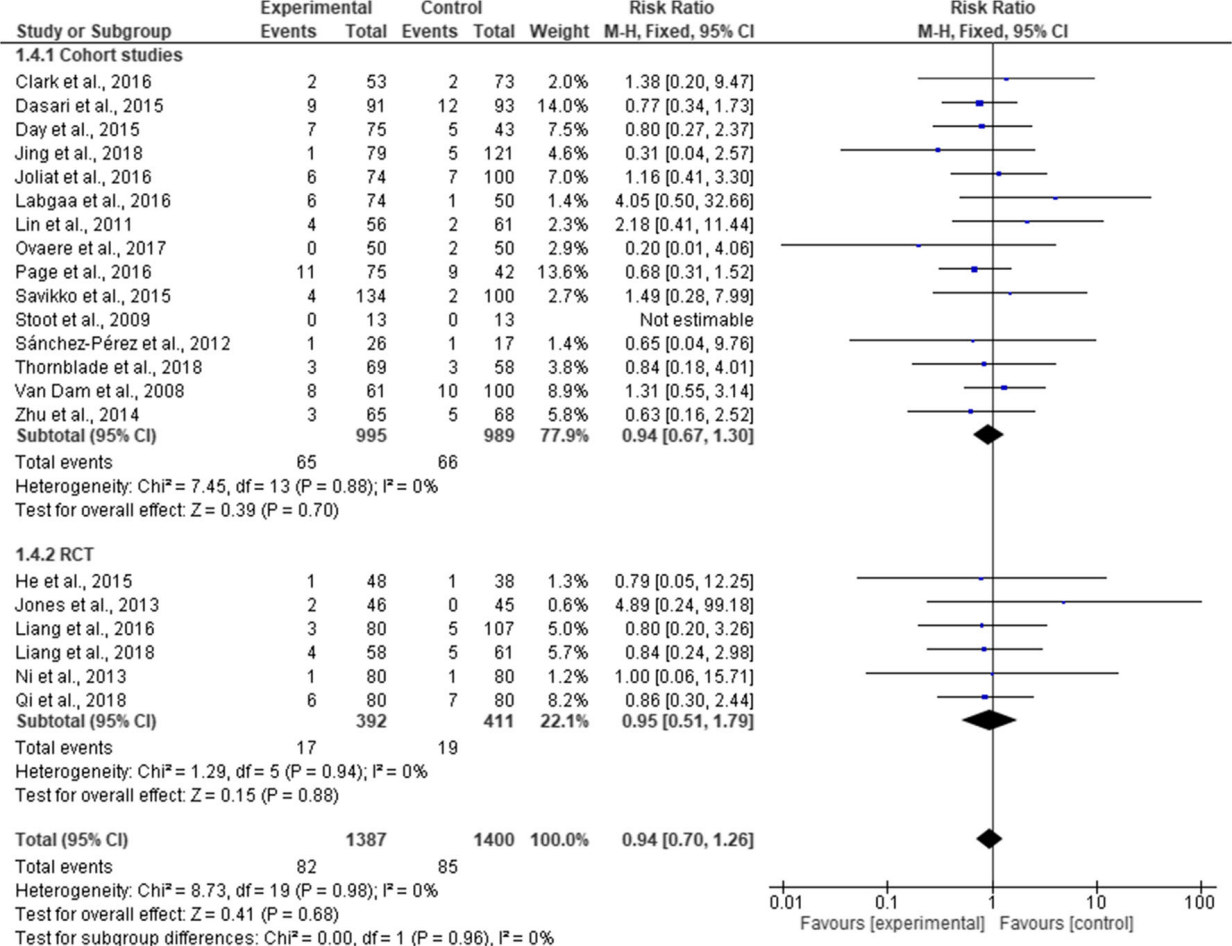
Forest plot of readmission rates, ERAS vs standard care; subgroup analysis (RCT & Cohort studies)

**Fig. 12:**
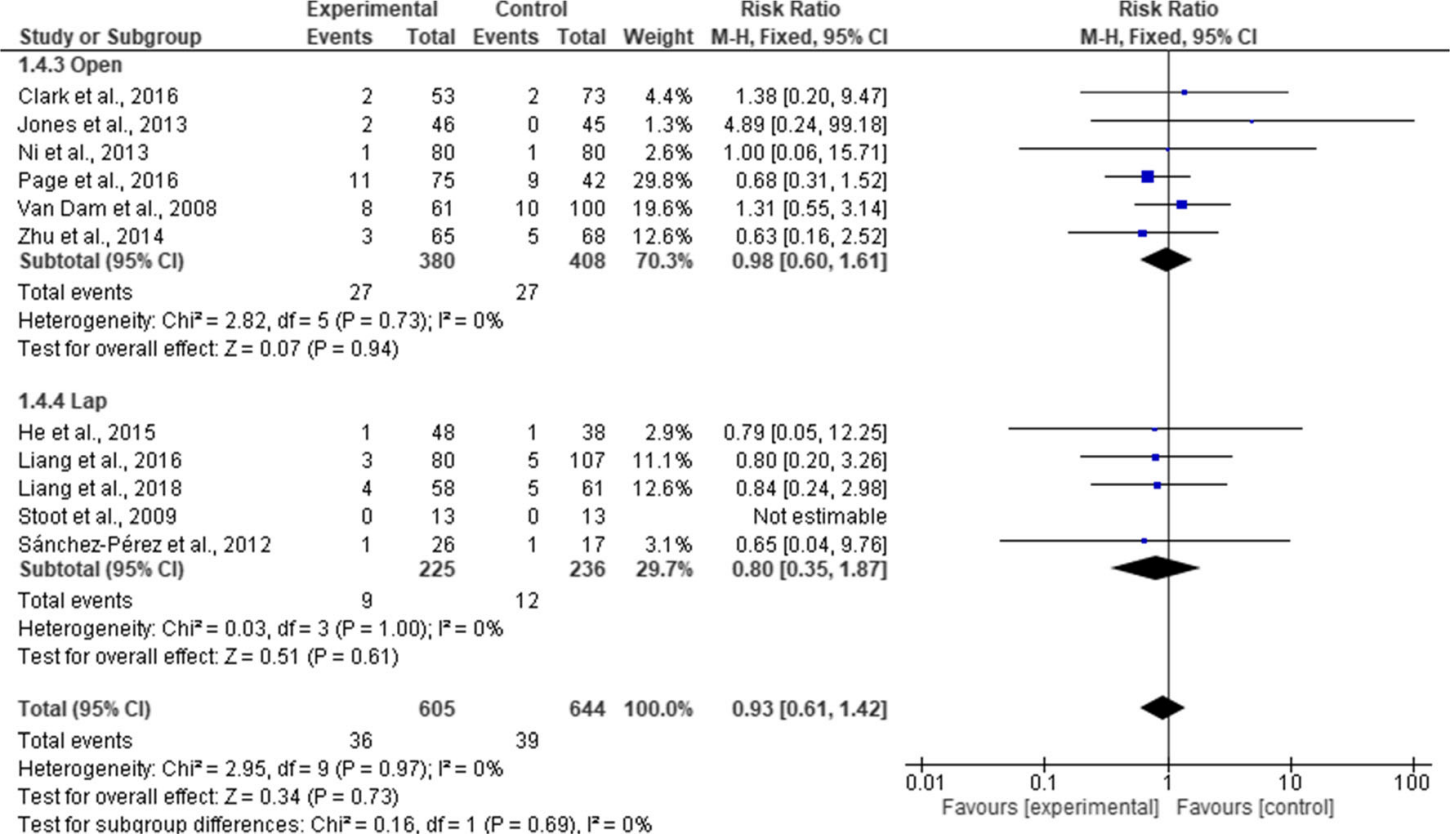
Forest plot of readmission rates, ERAS vs standard care; subgroup analysis (Open & Lap)

Twenty-one studies reported readmission rates (2878 patients). A total of 167 patients were readmitted (82 in ERAS group and 85 in standard care group). Pooling of the results, there was no significant difference in readmission rates between ERAS and standard care (RR, 0.94; 95% CI, 0.70–1.26; *p* = 0.68), with no significant heterogeneity observed in the studies (χ^2^ = 8.73; df = 19; *p* = 0.96; I^2^ = 0%). In the subgroup analysis, there was no significant difference in readmission rates between ERAS and standard care in both RCTs (RR, 0.95; 95% CI, 0.51–1.79; *p* = 0.88) and cohort studies (RR, 0.94; 95% CI, 0.67–1.30; *p* = 070). There is no significant difference in the overall readmission rates between RCTs and cohort studies (χ^2^ = 0.00; df = 1; *p* = 0.96; I^2^ = 0%) (Fig. 11). In a further subgroup analysis for the type of surgical approach, readmission rates were similar in the ERAS group and standard care for both open surgery (MD = 0.98; CI, 0.60 to 1.61; *p* = 0.94) and laparoscopic surgery (MD = 0.80; CI, 0.35 to 1.87; *p* = 0.61). There was no significant difference in the overall readmission rates between the two surgical approaches (χ^2^ = 0.16, df = 1 (*p* = 0.69), I^2^ = 0%) (Fig. 12).

### Mortality rates

**Fig. 13:**
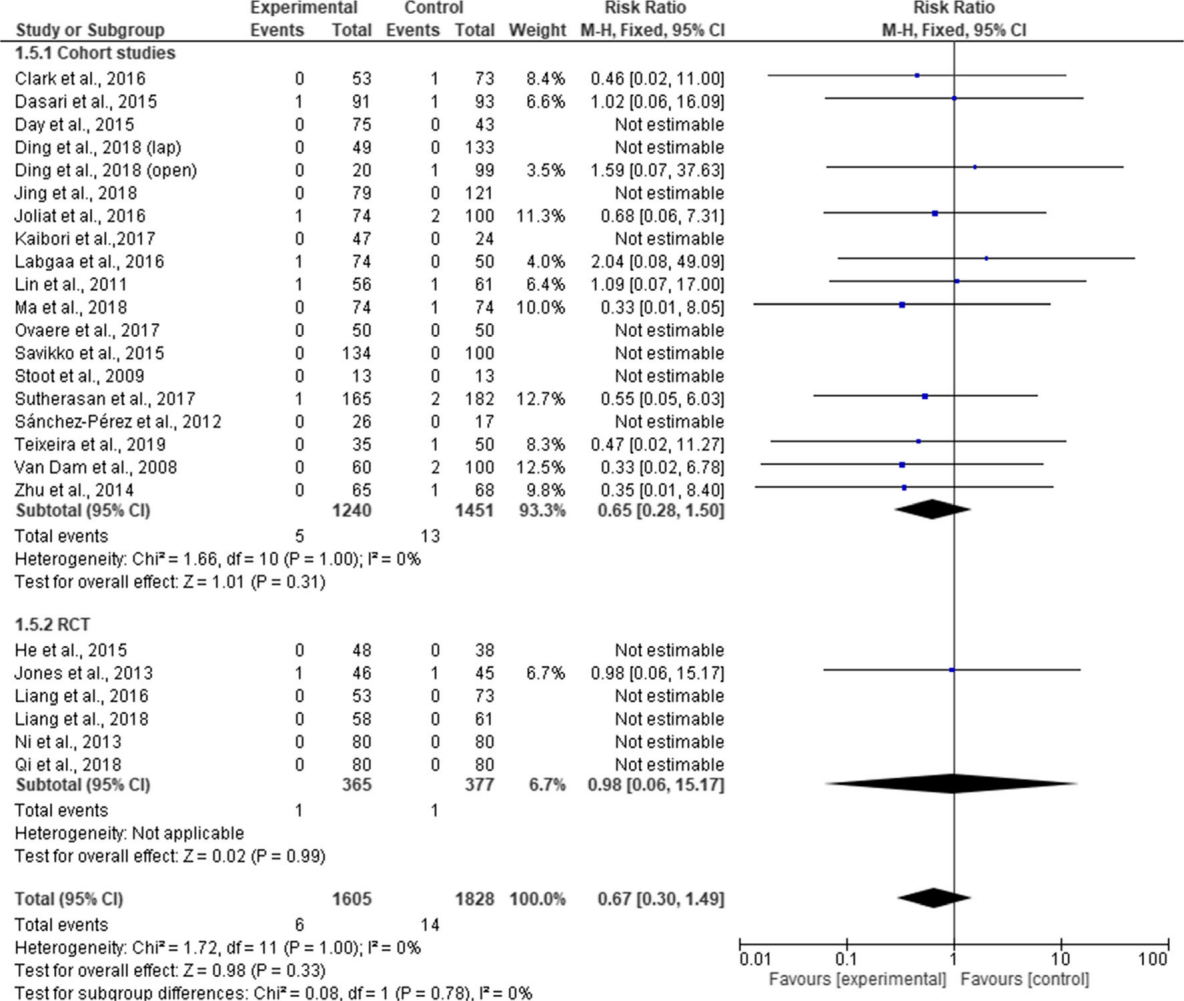
Forest plot of mortality, ERAS vs standard care; subgroup analysis (RCT & Cohort studies)

Twenty-five studies provided data on mortality rates (3433 patients). Of these studies, no mortality occurred in ten.^2, 18–20, 24–26, 30, 31, 39^ A total of 20 deaths were reported in the studies (6 in ERAS and 14 in standard care group). Pooling the results, there was no significant difference in mortality rates between ERAS group and standard care (RR, 0.67; 95% CI, 0.30–1.49; *p* = 0.33), and no significant heterogeneity observed among the studies (χ^2^ = 1.72; df = 11; *p* = 1.00; I^2^ = 0%). Subgroup analysis for RCT (RR, 0.98.; 95% CI, 0.06–15.17; *p* = 0.99) and cohort studies (RR, 0.65; 95% CI, 028. –1.50; *p* = 0.31) found no difference in the mortality rates between ERAS group and standard care group. There was no difference in mortality between the RCTs and cohort studies (χ^2^ = 0.08; df = 1; *p* = 0.78; I^2^ = 0%) **(**Fig. 13).

### Compliance

Four studies (474 patients) provided data on compliance to ERAS elements. Three of the four studies measured overall compliance to ERAS elements.^24, 27, 40^ Compliance was higher in the ERAS group across the three studies, ranging from 65% to 73.8% in the ERAS group and 20% to 48.7% in the standard group. Jones et al.^25^ reported 100% compliance in 18 out of the 19 ERAS elements in the ERAS group compared to 100% in 11 out the 19 ERAS elements in the standard care group.

## Discussion

This present review investigated the impact of ERAS programmes in liver surgery. A comprehensive search was performed resulting in 6 RCTs and 21 cohort studies with a total of 3739 patients, making this study the largest and most comprehensive review conducted on this topic to date. In addition to confirming that ERAS is safe and feasible, reduction of LOS and readmission rate without an increase in complications and mortalities in liver surgery,^7–14, 45, 46^ we extracted sufficient data to conduct a subgroup analysis in hospital cost and confirmed a significant reduction in the overall cost following the implementation of ERAS protocol in liver surgery.

Regarding the primary outcome, hospital costs were significantly lower in ERAS group in every study included in the meta-analysis (*p* < 0.0001). Whilst the main objective of ERAS protocol is not about healthcare cost savings, however, evident from this review confirms the results from two previous reviews^9, 10^ that introduction of ERAS protocol in liver surgery represents a significant reduction in hospital expenses. It was also evident that any significant reduction in length of hospital stay and complication rates was accompanied by significant reduction in total hospital expenses following implementation ERAS programme.

Our findings suggested that LOS was significantly reduced by 2.22 days in the ERAS group, which is similar to previous findings of others.^8–10, 12–14^ However, there was a significant heterogeneity in the analysis, which could not be eliminated even after conducting a sensitivity analysis. Moreover, the significant heterogeneity did not disappear even after two studies from Liang and associates which included patients less than 18 years old were removed from the analysis, neither did removing studies that reported median and range or applying a fixed model to the analysis eliminated the significant heterogeneity. One explanation for this variation could be due to the variation of the definition of LOS. Some studies measured total length of hospital stay, whilst others measured postoperative length of hospital stay after surgical intervention. Another possible reason for the variation was how discharge criteria were determined. Among the 17 studies that described discharge criteria ,^2, 19, 20, 24–33, 35, 37, 38, 41^ it became more apparent that there was no standardized discharge criteria for patients after liver surgery. Among all discharge criteria, the criteria suggested by Van Dam et al. (2008) including normal or decreasing serum bilirubin, good pain control with oral analgesia only, tolerance of solid food, no intravenous fluids, mobile independently or at the preoperative level and willingness to go home appear to be the most widely adopted by other liver units.

Similar to previous reviews,^8–10, 12, 13^ our findings again suggested a reduction in the overall complication rates in ERAS group compared to standard care, with a significant heterogeneity in the cohort studies. Contrary to the findings of Wang et al.,^10^ our results did not demonstrate a reduction in overall complication rates in open liver surgery. However, it is worth noting that this current review excluded studies that combined both minimal invasive and open surgical approach in the subgroup analysis. We believe that inclusion of these studies could introduce bias. Furthermore, our findings implied that complications in liver surgery are complex and cannot be reduced solely by ERAS pathway. Therefore, where possible, with the right technique and expertise, laparoscopic approach should be considered along with ERAS protocol to reduce risk of complications.

Regarding method of assessing complications, eight of the cohort studies did not use Clavien-Dindo or mentioned the method by which postoperative complications were evaluated,^2, 18, 20, 31, 34, 36, 39, 42^ suggesting Clavien-Dindo classification is the most common method of reporting and grading complications in liver surgery.

The cases of mortalities and readmissions were equally similar in the two groups (*p* = 0.68 and *p* = 1.00, respectively). The reason for this could be explained by the fact that incidents of readmissions and mortalities reported in the studies were too low in the studies to detect any significant differences. For example, overall cases of readmissions were 0.06% both in ERAS group and standard care group (82/1387 and 85/1400, respectively). Similarly, cases of mortality were very low across the studies, with 6 deaths (6/1605) in ERAS group and 14 deaths in standard care group (14/1828). Follow-up was 30 days across the studies with the exemption of three studies that reported a 90 days follow-up.^18, 19, 31^ These 3 studies have a combined total of 458 patients (218 ERAS and 240 standard care), with 4 deaths (1 in ERAS group and 3 in standard care group). Similarly, two studies reported 90 days readmission.^18, 19^ There was a 7.64% (11/144) readmission rates in ERAS group and 8.43% (14/166) in standard care group, respectively, which is similar to 5.9% (82/1387) overall average of readmission rates in ERAS group and 5.86% (82/1400) in standard care group, suggesting that 90 days follow-up may not be necessary.

There were significant variations in the number of elements utilized in each study. All studies applied 19 items or less with the exemption of Teixeira et al.^40^ that implemented the 23 elements as recommended in the current ERAS guidelines.^5^ The reason for this could be that ERAS protocols were implemented in these studies after the current guidelines were published.^5^ Elements commonly mentioned in the studies were preoperative education, no bowel preparation, minimal pre-op fasting, carbohydrate drinks, avoidance of excessive intravenous fluids, minimal use of nasogastric tube and abdominal drain, early resumption of normal diet and oral fluids, early removal of urinary catheter and enforced mobilization. Elements such as thromboembolism and antibiotic prophylaxis, short-acting anaesthetic agent, glycaemic control, prevention of postoperative nausea and vomiting and systemic audit were less commonly applied. There was notable disagreement on when to remove catheters and drains. For instance, catheters were removed within 24 h postoperatively in some studies, whereas it was left for up to postoperative day 3 in other studies (Table 2). Similarly, administration of oral analgesia was recommenced within 24 h in some studies, whilst in others, postoperation day 3 and 4 in one study. It appears that these decisions were down to surgeon preference rather than patients’ specific requirements, making the comparison among the studies impossible. Whether implementation of ERAS protocol will be successful or not largely depends on compliance. Conversely, compliance to ERAS elements was rarely measured in the included studies. Whenever compliance was measured, overall compliance remained was generally low even in the ERAS group. One way of measuring and monitoring compliance within ERAS programme is through systematic audit.^5^ However, only 18.5% (5/27) of the included studies reported performing a systematic audit^20, 24, 27, 28, 40^ to monitor of ERAS elements within the ERAS programme.

### Limitation

One of the key limitations in this study was the presence of significant heterogeneity in the effect of ERAS protocols on LOS and hospital cost. The studies included in this review were conducted in 11 countries across 4 continents (South America, North America, Europe and Asian). Most of these countries have difference healthcare system which may have contributed to the significant heterogeneity observed in the analyses. One notable example was the wide variation in the hospital cost reported across the studies. Although, a random effect was used when there was a significant evidence of heterogeneity, there was no guarantee that heterogeneity can be eliminated impacting on the validity on our results. The majority of studies included in this review were conducted over a period of time when ERAS protocols were not standardized or fully established, with a wide variation in the elements applied in the protocols. Therefore, it is not clear whether this has introduced bias regarding the exact impact of ERAS protocols compared to standard care. Similarly, most of the included studies were based on retrospective data analysis introducing further bias to this review. Furthermore, the majority of the cohort studies were low-quality with small and unequal sample sizes which may lead to confounding bias. A larger high-quality multicentre and multinational RCTs is recommended to confirm some of the findings in this review.

## Conclusion

Our review concluded that the introduction of ERAS protocols is safe and feasible in hepatectomies, without increasing mortality and readmission rates, whilst reducing LOS and risk of complications, and with a significant hospital cost saving. Laparoscopic approach may be necessary to reduce complication rates in liver surgery. However, further studies are needed to investigate overall compliance to ERAS protocols and its impact on clinical outcomes.
